# Microtomographic, Histomorphological, and Histomorphometric Analysis of Bone Healing in the Midpalatal Suture After Treatment with Isotretinoin

**DOI:** 10.3390/dj13040142

**Published:** 2025-03-25

**Authors:** Maria Júlia Bento Martins Parreira, Daniela Vieira Buchaim, Ana Carolina Cestari Bighetti, Marcos Antonio Girotto, Miguel Ângelo de Marchi, Dayane Maria Braz Nogueira, Augusto Alberto Foggiato, Juliana Zorzi Coléte, Acácio Fuziy, Rogerio Leone Buchaim

**Affiliations:** 1 Postgraduate Program in Applied Dental Sciences, Bauru School of Dentistry (FOB/USP), University of Sao Paulo, Bauru 17012-901, Brazil; mjuliaparreira@usp.br (M.J.B.M.P.); anacarolinacb@usp.br (A.C.C.B.); dayanenogueira@usp.br (D.M.B.N.); 2 Graduate Program in Anatomy of Domestic and Wild Animals, Faculty of Veterinary Medicine and Animal Science (FMVZ/USP), University of Sao Paulo, Sao Paulo 05508-270, Brazil; danibuchaim@alumni.usp.br; 3 Anatomy Department, Medical School, University Center of Adamantina (FAI), Adamantina 17800-000, Brazil; 4 Multiprofessional Preceptorship for Undergraduate Health Studies, Faculty of Medicine of Bauru (FMBRU-USP), University of Sao Paulo, Bauru 17012-901, Brazil; 5 Oral Health Coordination, Municipal Health Department, Marília 17501-002, Brazil; drmarcosgirotto@gmail.com; 6 Dermatology Department, Medical School, University Center of Adamantina (FAI), Adamantina 17800-000, Brazil; mamarchi@terra.com.br; 7 Department of Radiology, Pediatrics, and Orthodontics, State University of Northern Paraná (UENP), Jacarezinho 86400-000, Brazil; augusto.foggiato@uenp.edu.br; 8 Department of Oral and Maxillofacial Surgery, Traumatology, and Stomatology, State University of Northern Paraná (UENP), Jacarezinho 86400-000, Brazil; juliana.zorzi@uenp.edu.br; 9 Specialization Program in Orthodontics, Brazilian Dental Association (ABO), Goiânia 74325-110, Brazil; acacio.fuziy@uenp.edu.br; 10 Department of Biological Sciences, Bauru School of Dentistry (FOB/USP), University of Sao Paulo, Bauru 17012-901, Brazil

**Keywords:** isotretinoin, bone repair, bone, bone regeneration, palatal expansion technique, rats, retinoid, vitamin A, orthodontic appliance design, acne

## Abstract

**Background/Objectives:** Rapid palatal expansion is commonly used to correct maxillary deficiencies. However, medications like Isotretinoin may influence bone remodeling during treatment. Isotretinoin, a drug used to treat acne, was the focus of this study, which aimed to evaluate its effect on bone repair after rapid palatal suture expansion in rats. **Methods:** The sample consisted of 40 Wistar rats, divided into two groups: the control group (CG), subjected only to orthodontic movement, and the experimental isotretinoin group (IG), where movement occurred after drug administration. Periods of 0, 7, and 14 days after the installation of the orthodontic device were analyzed, with a force of 30 g applied in all groups using a steel spring. After euthanasia, the maxillae were removed and analyzed via Micro-CT, histologically, and histomorphometrically. **Results:** The results showed that the expander was effective in promoting the expansion of the palatal suture. After 14 days, the total expansion was 381% (CG) and 299% (IG); the percentage of vessels in the connective tissue increased by 145% in CG and 84% in IG; and bone formation in both groups occupied 52% of the expanded palatal suture. No significant differences were observed between the groups regarding collagen fiber formation. **Conclusions:** It was concluded that the daily administration of Isotretinoin at the standard dose for the treatment of severe acne does not cause significant alterations in the bone repair process following the opening of the median palatine suture in rats.

## 1. Introduction

A procedure widely used to improve the transverse dimension in patients with maxillary deficiencies is rapid maxillary expansion by opening the midpalatal suture. During the procedure, the suture undergoes remodeling, which includes bone resorption and the formation and rearrangement of type I collagen fibers [1,2]. During the treatment of this malocclusion, the patient may be taking certain medications that can interfere with orthodontic movement and accelerate bone formation in the expansion region, which is of the utmost importance to prevent relapse [3].

One of the most commonly prescribed medications is 13-cis-retinoic acid, also known as Isotretinoin and commercially branded as Roaccutane/Accutane^®^ (Catalent Germany Eberbach GmbH, Eberbach, Germany). This compound, a derivative of vitamin A, functions by regulating biological processes in various cell types, either directly or indirectly. Isotretinoin has been extensively utilized in the treatment of severe acne [4,5,6].

According to Layton (2009), a dose of 0.5–1.0 mg/kg/day drastically reduces sebum excretion by about 90% within 6 weeks. The majority of patients taking oral Isotretinoin will be acne-free after 4 to 6 months of treatment, depending on the prescribed dosage [7]. According to the literature, the prolonged use of Isotretinoin can lead to a decrease in bone mineral density, increasing the risk of fractures and osteoporosis, as well as causing bone alterations in young individuals during their development [8,9,10,11,12,13,14]. Additionally, many studies highlight that the effects of Isotretinoin on bone density and bone growth depend on the dose and duration of treatment and are therefore directly related to the use of high doses for a prolonged period [15,16,17].

The interference of Isotretinoin in bone remodeling can be explained, for example, by the inhibition of osteoblastic activity and increased bone resorption. Even in osteogenic conditions, with increased osteoblastic differentiation by the experimental use of BMP2, retinoids may not inhibit alkaline phosphatase (ALP) activity, but they affect cell morphology, by interfering with bone anabolism. Overall, these in vivo studies demonstrate that vitamin A inhibits cortical bone formation and does not always affect trabecular bone formation [18,19]. Bone tissue, as well as cartilage, presents regulatory effects and interactions in signaling systems. The Wnt and OPG-RANKL-RANK systems, as key mediators, interact in bone remodeling. Under experimental conditions of abnormal mechanical loading, the pro-inflammatory environment and lower OPG/RANKL ratio trigger apoptosis and matrix loss, due to the action of metalloproteinases, such as MMP-13 or MMP-9 [20,21].

It is known that excessive intake of vitamin A leads to considerable adverse effects on the skeleton [22], and knowing whether patients who use Isotretinoin are fit to undergo orthodontic treatments is essential for the execution of the treatment in a controlled manner. It is worth noting that the phase in which mineral bone is formed and its storage maximized is during adolescence, the same period in which patients often use Isotretinoin to treat severe acne [23,24].

Taking into account that previous research has shown contradictory results on the effect of this substance, Isotretinoin, on bone metabolism and the healing of gingival wounds [25,26], i.e., both adverse effects such as hyperostotic changes, the premature closure of epiphyses, an increased number and size of osteoclasts, and decreased osteoid surface, and benefits such as increased bone formation in calvaria and dental alveoli [27], and considering that the literature is scarce in the analysis of the effects of this drug in conjunction with maxillary expansion [28,29,30], we sought to overcome a remaining gap in the literature using previously unperformed evaluations, such as Micro-CT, Masson’s trichrome staining, and the analysis of collagen fibers by Picrosirius-red polarization method, all performed in our study.

In view of the above, this preclinical study aimed to evaluate the influence of this drug (Isotretinoin) on bone repair, after opening the median palatine suture in rats, through microtomographic (Micro-CT), histomorphological, and histomorphometric analysis.

## 2. Materials and Methods

### 2.1. Ethical Aspects and Experimental Design

This preclinical study was approved by the Animal Use Ethics Committee (CEUA) of the University of Marília (UNIMAR), registered under number 16/2018. Forty male Wistar rats (*Rattus norvegicus*), aged 12 weeks and weighing an average of ±345 g, provided by the Central Bioterium of the University of Marília (UNIMAR, Marília, Brazil), were used.

The animals were kept under a 12 h light/dark cycle and a controlled temperature of 23 °C. They were fed Labina feed (Purina^®^, São Paulo, Brazil). The study was conducted in accordance with the guidelines of the Declaration of Helsinki, the ARRIVE (Animal Research: Reporting of in vivo Experiments) guidelines, and the NC3Rs (National Centre for the Replacement, Refinement & Reduction of Animals in Research) principles. Throughout the experimental phase, the animals were monitored by the researchers for possible altered habitual behavioral characteristics [31].

**Figure 1:**
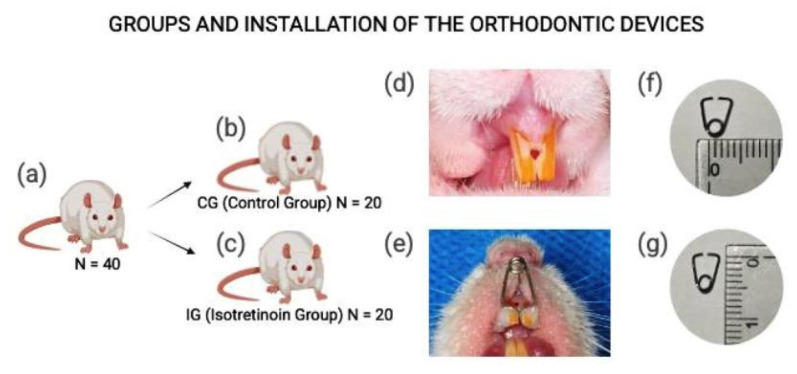
Groups and installation of the orthodontic devices (**a**–**g**): (**a**) initial sample; (**b**) control group (CG); (**c**) Isotretinoin group (IG); (**d**) orifice for the installation of the orthodontic device; (**e**) properly activated and installed orthodontic device; (**f**) diameter of the helix; (**g**) length of the orthodontic device.

The animals were divided into 2 groups: a control group (CG), where the animals were subjected only to orthodontic movement, and an experimental isotretinoin group (IG), where the induced tooth movement occurred after drug administration. The periods of 0 days (day of spring installation), 7 days, and 14 days after the placement of the orthodontic device were analyzed (Figure 1).

### 2.2. Drug Treatment

Isotretinoin was obtained from the drug Roaccutane/Accutane^®^ (Catalent Germany Eberbach GmbH, Eberbach, Germany), and the daily dose administered was 7.5 mg/kg, chosen by our group of researchers based on similar, previously published studies in the field [4,26,28,32,33]. Generally, the recommended daily dose for humans can vary from 0.5 to 2 mg/kg/day [34]. Therefore, in the dosage and dosage adjustment between humans and animals, we chose 7.5 mg/kg/day. This dose can promote serum levels of the drug in rats compatible with those of humans, corresponding to the dose of 1 mg/kg of body weight [32].

The medication was administered orally to the animals in IG, using the gavage technique, for a period of 30 days, before the installation of the orthodontic device. The animals in CG received water to simulate the same stress as in IG. After the installation of the devices, the animals continued to receive the medication until the 14th day, corresponding to the second period of euthanasia.

### 2.3. Installation of the Orthodontic Device (Expander)

To install the device, the animals were anesthetized with an intramuscular injection of 50% tiletamine and 50% zolazepam (Zoletil 50^®^; Virbac Brazil, São Paulo, Brazil) at a dose of 3 mg/kg of body weight [35,36]. The spring was made with 0.18 stainless steel wire (Orthometric^®^, Marilia, Brazil) and previously activated extraorally, producing a force of 30 g [37,38,39,40,41,42]. The spring was inserted into the upper central incisors through a hole made in the midline with a 1011 diamond bur (KG Sorensen^®^, Serra, Brazil) and fixed with Opallis Flow resin (FGM^®^, Joinville, Brazil). The devices were not reactivated until the animals were euthanized, which occurred 7 and 14 days after their installation.

### 2.4. Maxillary Collection and Microtomographic Evaluation

Euthanasia was performed using a lethal dose of sodium pentobarbital (Thiopentax^®^, Cotia, Brazil), at 150 to 200 mg per kg of body weight [35,36]. Subsequently, the maxilla of each animal was removed, dissected, and fixed in 10% formaldehyde phosphate buffer (pH 7.4) for 48 h.

All samples were scanned in an X-ray computerized microtomographer (Skyscan model 1272, Bruker-Micro-CT^®^, Kontich, Belgium). The region of interest (ROI) started from the middle palatal bone margins and extended bilaterally on both sides. The device was set at 70 kV and at 142 uA [43]. A filter was used (0.5) and the sample was rotated 180 degrees with a rotation step of 0.5, generating an acquisition time of 41 min per sample. The NRecon program with its software package (Bruker, Belgium) was used to reconstruct the three-dimensional images. Posteriorly, the images were aligned in the coronal, transaxial, and sagittal planes using the DataViewer program. As suggested by Nascimento et al. (2024) [44], linear measurements of the palatal suture were obtained in the axial view at four distinct and equidistant points (P1, P2, P3, and P4) by tracing bilateral bony edges. In addition, the inter-incisor distance (DI) was measured at the closest approximation point of the dental elements.

### 2.5. Histomorphometric Evaluation

The samples were decalcified with 4.13% ethylenediaminetetraacetic acid (Titriplex III—Merck KGaA^®^, Damstadt, Germany) plus 0.44% sodium hydroxide for 30 days. The specimens were histologically processed for embedding in polymer-enriched paraffin Histosec (Merck KGaA^®^, Damstadt, Germany). Coronal sections of 5-µm thickness of each point (P1, P2, P3, and P4) were obtained and stained with hematoxylin and eosin (HE), Masson–Goldner trichrome, and Picrosirius-red. All histological slides were coded according to experimental groups. An experienced examiner evaluated them blindly.

Virtual microscopy, data management, and image analysis were conducted using the Aperio ScanScope system (Leica Biosystems Imaging^®^, Nussloch, Germany). Each section of palatine process was captured using an Aperio CS2 scanner (Leica Biosystems Imaging^®^, Nussloch, Germany), with an original magnification of ×20. Subsequently, the digital images were analyzed using Aperio ImageScope v.12.4.3.5008 software (Leica Biosystems Imaging^®^, Nussloch, Germany). The sections stained with HE and Masson–Goldner trichrome were used for morphological description and linear measurements of the palatal suture, as well as the percentages of new bone and soft tissue in the midpalatal suture. The percentage of blood vessels in the connective tissue (vessels area/total area of the connective tissue) and the midpalatal suture also were determined. This analysis was performed by a single experienced investigator (ACCB).

To assess the collagen structure of the palatine suture, we performed Picrosirius-red staining as described by Bighetti et al., 2024 [45]. The palatal suture was subjected to polarized light. All images were acquired under identical camera settings, including white balance, gain, and exposure. The exposure time was selected to limit the occurrence of intensity saturated pixels. A total area of 144 × 10^6^ pixels^2^ was evaluated for each sample. The area of birefringent fibers (in pixels^2^) of the palatal suture and each polarization color were determined using Leica Application Suite X software (Leica Microsystems^®^, Heerbrugg, Switzerland). Under polarized light, the larger collagen fibers are bright yellow or red, and the thinner ones, including reticular fibers, are green.

### 2.6. Statistical Analysis

The results obtained in the linear measurements at the midpalatal suture and DI and histomorphometric parameters were submitted to a Shapiro–Wilk normality test prior to statistical tests, to verify the values’ normality. Subsequently, the data were evaluated using the one-way ANOVA test and Tukey’s post hoc test to explore differences between means among periods, and Student’s *t*-test was used to compare the means between two groups in each period. We used Statistica v.10.0 software (StatSoft Inc. ^®^, Tulsa, OK, USA), with a level of significance of 5%. Continuous variables are shown with the absolute mean ± standard deviation (SD).

## 3. Results

The descriptive statistical analyses and their normality, for all variables included in the objectives of this study, demonstrated that all of them are normal (*p* > 0.05), according to the significance for Shapiro–Wilk (Table S1, Supplementary Materials).

### 3.1. Linear Measurements of Palatal Expansion in Microtomographic Images

**Figure 2:**
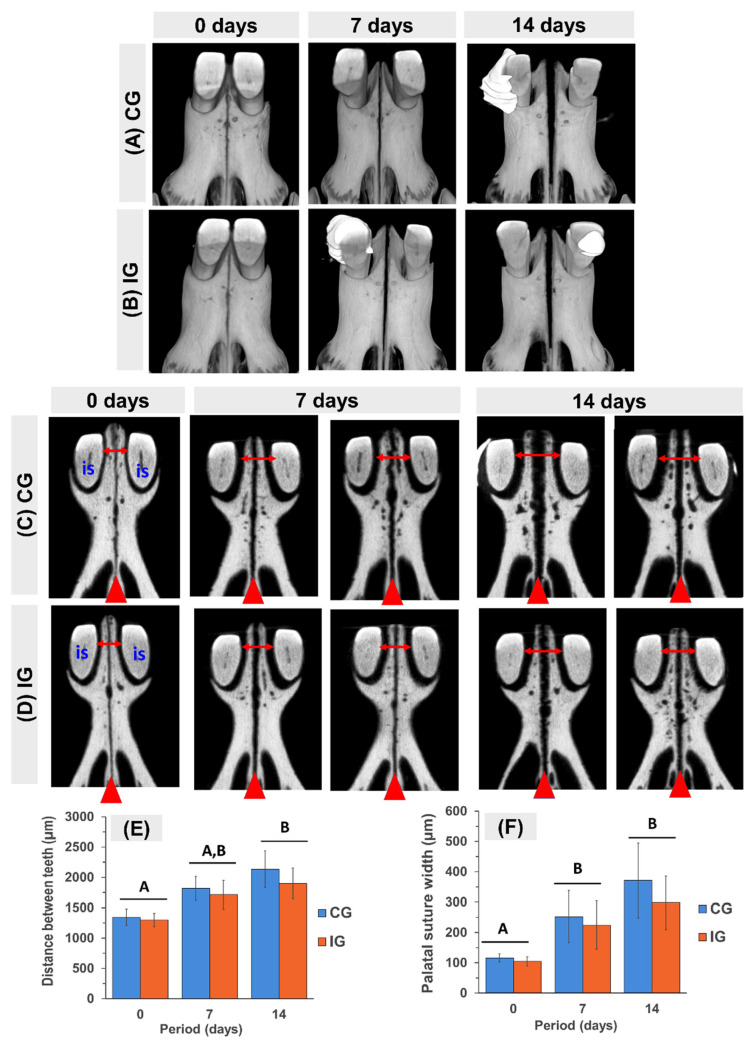
Micro-CT results of rapid maxillary expansion in rat at 0 days (pre-expansion) and 7 and 14 days post-expansion. (**A**–**D**) Three-dimensional Micro-CT views (**A**,**B**) and two-dimensional Micro-CT views (**C**,**D**) of the anterior palatal plate part of the maxilla, showing the natural positioning of the incisors (is) and median palatine suture (red arrowhead) at 0 days. It can be noticed that at 7 and 14 days, the distance between the incisors (double red arrow) increases and changes in the positioning/inclination of the incisors are observed. Note the gradual increase in palatal suture width (red arrowhead). (**E**,**F**) Bar graphs of mean ± SD of distance between teeth (**E**) and palatal suture width (**F**). Groups with different letters are significantly different; *p* < 0.05 (A ≠ B).

Three-dimensional (3D) and two-dimensional (2D) microtomographic images of the palatine region at 0 days (pre-expansion) and 7 and 14 days post-expansion are shown, respectively, in Figure 2A,B and 2C,D. The intraoral self-activated expander with an active helix of 1.5 mm in diameter exerted 30 g of force, which was enough to displace central incisors laterally without additional activation. At 0 days (pre-expansion), the distance between incisive (Figure 2E) and palatal suture width (Figure 2F) did not show statistical difference between groups (*p* > 0.05), presenting an average value of 1319 µm and 110 µm, respectively. The expansion was successful in all the animals, with a mean separation of the central incisors of 2134 µm (an increase of 58.9%, *p* < 0.001) in CG and 1802 µm (an increase of 46.5%) in IG at 14 days. However, changes in the positioning/inclination of the incisors were also observed. In this period, the palatal suture width increased 220% (an increase of 225 µm; *p* = 0.00018) in CG and 185.3% (an increase of 193 µm; *p* = 0.00023) in IG. Lower values of palatal suture width were obtained for IG compared to CG, but no statistical differences were seen between them (*p* = 0.40645).

### 3.2. Descriptive Histology and Histomorphometry

**Figure 3:**
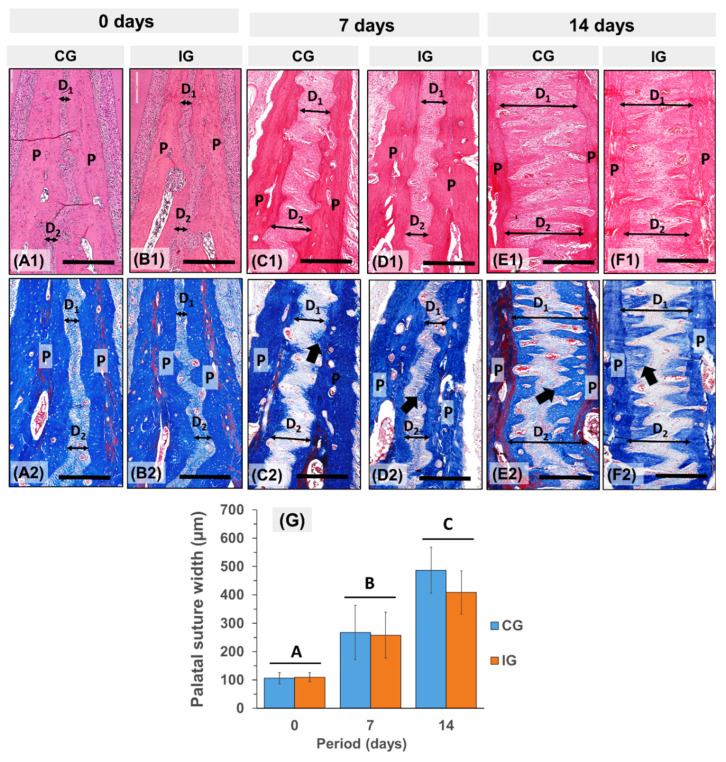
Histomorphometric results of rapid maxillary expansion in rat at 0 days (pre-expansion) and 7 and 14 days post-expansion. (**A**–**F**) Panoramic histological view of hematoxylin/eosin (**A1**–**F1**)- and Masson trichrome (**A2**–**F2**)-stained sections at 0 days (**A1**,**A2**), showing a small distance (**D1**,**D2**) between the palatine processes (P) of maxillae. Note that between 7 (**C1**,**D1**,**C2**,**D2**) and 14 days (**E1**,**F1**,**E2**,**F2**), the distance between palatine processes (D1 and D2), as well as the bone formation (black arrow) in their surfaces, increased. Scale bar = 400 µm. (**G**) Bar graphs of mean ± SD of palatal distance. Groups with different letters are significantly different; *p* < 0.05 (A ≠ B ≠ C).

At 0 days (pre-expansion), in both groups, the palatine suture was composed by fibrocellular connective tissue that connect bone to bone, with the presence of many small vessels and the absence of inflammatory infiltrate. In addition, small and sparse areas of newly formed bone on the margins of the suture ends were observed. As an observation in Micro-CT analyses, the suture width was similar in both groups (*p* = 0.9425; compare Figure 3A1,A2 with Figure 3B1,B2), with an average of 109 µm (Figure 3G).

**Figure 4:**
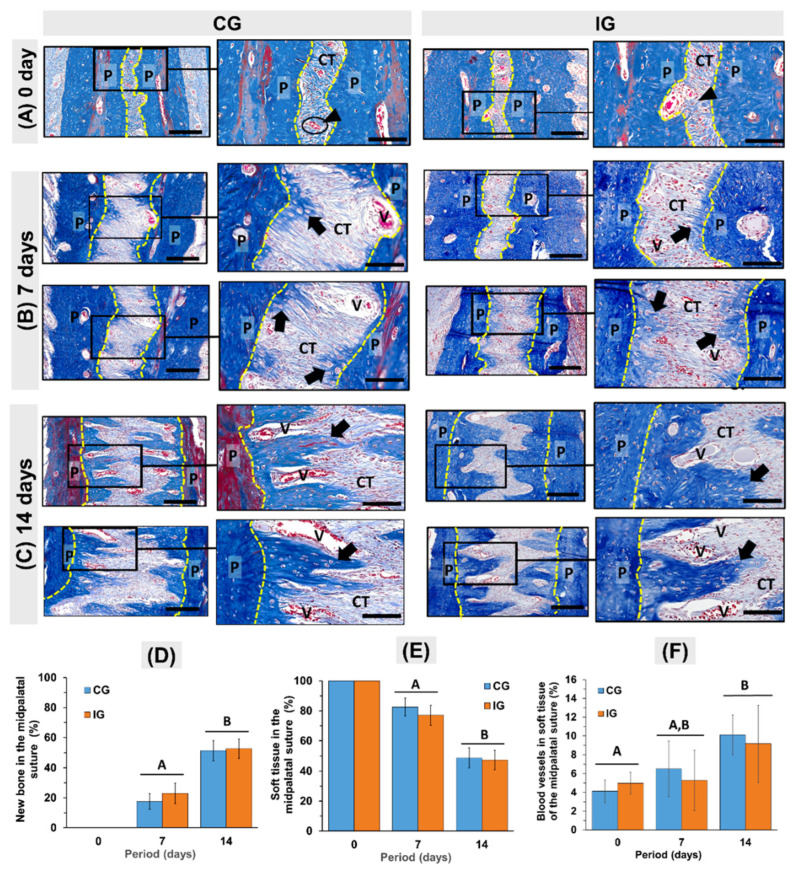
Bone formation during rapid maxillary expansion in rat at 0 days (pre-expansion) and 7 and 14 days post-expansion (hematoxylin/eosin). (**A**) At 0 days, the median palatine suture (the area between the yellow dotted lines) between the right and left palatine bones (P) was occupied by organized dense collagen fibers (CT) and some vessels (black arrowhead) between palatine processes (P). (**B**) At 7 days, the distance between the palatine processes increased and newly formed spicules of bone (black arrow) can be seen on the surface of the palatine bones (P). (**C**) At 14 days, the bony spicules from suture margins grew toward the center of the expanded midpalatal suture. Between the bony spicules, large blood vessels (V) can be observed. Masson’s staining. Scale bars of 100 µm and 200 µm (detail). (**D**,**E**) Bar draphs of mean ± SD of new bone (**D**) and soft tissue (**E**) in the midpalatal suture, and blood vessels in the connective tissue of the midpalatal suture (**F**). Groups with different letters are significantly different; *p* < 0.05 (A ≠ B).

Under the expansive force, the midpalatal suture was widened and bone formation was observed in both groups. At 7 days, the palatal suture width increased on average 118% (an average of 238 µm, *p* < 0.0029). The collagen fibers appeared stretched, and vessels increased in the caliber, leading to the extravasation of blood cells into the adjacent tissue. In this period, the stretched blood vessels from the bone walls observed along the stretched fiber bundles were remarkable. Active osteoblasts and new bone matrix deposition were present on the surface of the palatal bones (Figure 3C2,D2 and Figure 4B). It was possible to observe a reversion line that delimited the presence of the formation of a new bone band that permeated the suture area. On day 7, the new bone tissue occupied 19.8% and 23% of the palatal suture in CG and IG, respectively (Figure 4B). There was also the presence of occasional osteoclasts associated with small areas of bone resorption.

At the end of day 14, histologically, the total palatal expansion in CG and IG was 381% (*p* = 000178) and 299% (*p* = 000178), respectively (Figure 3G). The bone formation that began on the surface of the palate grew in the form of spicules towards the center of the suture (Figure 3E1,E2,F1,F2 and Figure 4C). In this period, the bone formation in both groups (*p* < 0.05) occupied 52% of the expanded palatal suture and 48% by connective tissue (Figure 2E). Between 0 and 14 days, the percentage of vessels in connective tissue increased 145% (4% at 0 days vs. 10% at 14 days, *p* < 0.00957) in CG and 84% (5% at 0 days vs. 9.2% at 14 days, *p* < 0.00313) in IG.

### 3.3. Quantification of Collagen Fibers by Picrosirius-Red Polarization Method

**Figure 5:**
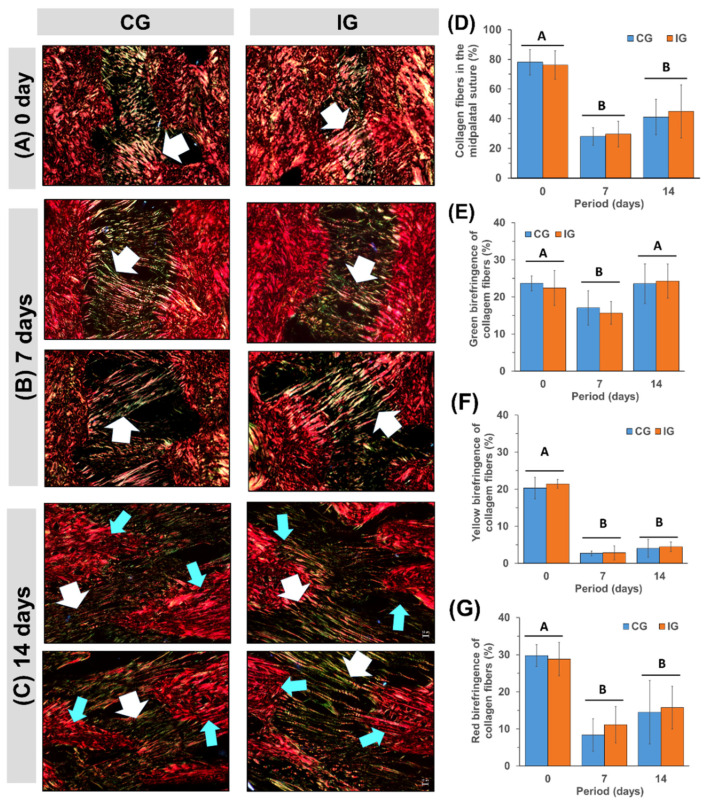
Polarized collagen fibers of palatal suture stained with Picrosirius-red during rapid maxillary expansion in rat at 0 days (pre-expansion) and 7 and 14 days post-expansion. (**A**) At 0 days, the midpalatal suture shows orange/red dense/thick collagen fibers (white arrow). (**B**) On day 7, the collagen fibers (white arrow) were thinner and greener compared to day 0/baseline. On day 14 (**C**), the expanded midpalatal suture showed interdigitated bone spicules (blue arrows) from the suture margins. The right and left bone spicules are interconnected by a mixture of thin/green and/or thick/yellow collagen fibers (white arrow). Birefringence quantifications of collagen fibers are represented in the bar graphs of the mean ± SD of collagen fiber percentages in the midpalatal suture (**D**) and the percentage of red (**E**), yellow (**F**), and green (**G**) birefringence fibers. Groups with different letters are significantly different; *p* < 0.05 (A ≠ B).

On day 0 (pre-expansion, Figure 5A), the palatal suture of CG and IG stained with Picrosirius-red under polarization microscopy exhibited on average 76% of collagen fibers (Figure 5D). In this method, the color of collagen fibers depends on fiber thickness. On day 0, the percentage of green polarization colors, indicating fine/immature fibers, was 24% (34.8 × 10^6^ pixels^2^, Figure 5E), with yellow color, denoting intermediate fibers, at 22% (31.5 × 10^6^ pixels^2^, Figure 5F) and red color, corresponding to thick/mature fibers, at 31% (44 × 10^6^ pixels^2^, Figure 5G).

During palatal expansion, the birefringence of collagen fibers showed no statistical difference between groups (*p* = 0.96731 for comparison at 0 days; *p* = 0.41466 at 7 days; *p* = 0.27979 at 14 days). However, a significant difference was observed between periods. Between 0 and 7 days, the collagen fibers decreased sharply, from 110.6 × 10^6^ pixels^2^ to 41.7 pixels^2^ (*p* < 0.0274), due to the stretching of collagen fibers, which generates large spaces occupied by interstitial fluid between them (compare Figure 5A with Figure 5B). With the stretching of the fibers, we observed a large reduction in intermediate and thick fibers. The reduction in yellow birefringent fibers in the cleft palate was 87% (from 31.5 × 10^6^ pixels^2^ to 27.5 × 10^6^ pixels^2^; Figure 5F), followed by a 68% reduction in red birefringent fibers (from 44.3 × 10^6^ pixels^2^ to 14 × 10^6^ pixels^2^; Figure 5E) and 29% in green birefringent fibers (from 34.9 × 10^6^ pixels^2^ to 23.2 × 10^6^ pixels^2^; Figure 5G). From 7 to 14 days, palatal expansion was still in progress with the stretching of the collagen fibers and extensive new bone formation on the surface of the palatine bones (Figure 5C). The collagen fibers went from 41.7 × 10^6^ pixels^2^ at 7 days to 62.2 × 10^6^ pixels^2^ at 14 days (*p* < 0.0194 for CG; *p* = 0.02014 for IG). On day 14, yellow (*p* = 0.80912 for *CG*; *p* = 0.12861 for *IG*; Figure 5E), and red birefringent fibers (*p* = 0.05171 for *CG*; *p* = 0.30189; Figure 5F) were like those observed at 7 days, while an increase of 47% (*p* = 0.00334 for CG; *p* = 0.018701 for IG) in green birefringent fibers (Figure 5D) was observed.

## 4. Discussion

The present study was carried out with the objective of evaluating the quality of newly formed bone after the expansion of the median palatine suture, under the influence or not of the drug Isotretinoin. It can be observed that the expansion of the maxilla was effective; however, Isotretinoin did not harm the percentage and quality of the new bone formed in the dosage we used.

When we compared CG with IG, in each of the periods evaluated, there was no significant difference between them. According to Figure 3, the histological analysis of sections stained with hematoxylin and eosin and Masson’s Trichrome at 0 days (A1–A2) show a small distance (D1 and D2) between the palatine processes (P) of the maxillae. In the periods of 7 (C1–D1 and C2–D2) and 14 days (E1–F1 and E2–F2), the distance between the palatine processes (D1 and D2) increased. Likewise, there was an increase in bone formation (black arrow) on their surfaces.

When comparing the average distance between the teeth of the two groups (CG and IG) between the periods, there was no significant difference on day 7 in relation to the others, but there was a significant difference between day 0 and day 14. This fact proves that the expansion was effective and was greater on day 14 in relation to day 0.

As stated in the research conducted by Ranjan et al. (2014), staining with Picrosirius-red allows for the evaluation of collagen maturity using polarized microscopy. The color and birefringence of collagen fibers depend on their thickness and molecular organization, with thin fibers ranging from green to yellowish-green and thick fibers from orange to red [46]. The predominant presence of green indicates lightly compacted collagen, which may favor tumor progression. This color pattern is associated with various pathological conditions, such as tumors of the salivary glands, thyroid carcinomas, and odontogenic cysts [47,48].

According to the literature findings that state that Isotretinoin, as a derivative of vitamin A, has adverse effects like those of hypervitaminosis A [49,50,51], it was expected that Isotretinoin would have a negative impact on bone repair after the opening of the palatine suture. However, the results of the present study indicated that bone repair after rapid maxillary expansion does not represent a problem with the concomitant use of Isotretinoin, since there was no significant difference in bone repair between CG and IG in any of the evaluations performed.

The results obtained contradict the results obtained by Hotchkiss et al. (2006) [52], who reported significant adverse effects on bone using the same dose of Isotretinoin in rats, and the results of Leachman et al. (1999) [49], where they measured bone density and calcium metabolism in men, aged between 17 and 25 years, receiving oral Isotretinoin for the treatment of acne and a group of healthy volunteers, aged between 19 and 26 years. Comparing the individuals treated with Isotretinoin and the healthy control individuals, the mean bone density was lower in all sites (spine, femur, and Ward’s area). Thus, they concluded that a loss of bone density is probably a direct effect of retinoids on bone.

The results presented here are in line with the findings of Tekin et al. (2008) [53], who investigated 36 patients who used Isotretinoin to treat severe acne for a period of 4 to 6 months. It was observed that a single treatment of Isotretinoin has no clinically significant effect on bone metabolism, as there is no detectable change in radiographic examinations. The present results are also consistent with the study by Nishio et al. (2017) [26], who carried out a study with 60 rats evaluating the effect of retinoic acid on tooth movement and periodontal healing after tooth extraction. The animals in the experimental group received Isotretinoin for 37 days at a concentration of 7.5 mg/kg and the animals in the control group received oil. The authors concluded that Isotretinoin did not affect orthodontic movement nor caused changes in maxillary bone volume, but that it may contribute to the acceleration of gingival healing.

Bulut et al. (2020) [28] also conducted a study where they examined the effects of Isotretinoin on bone formation after palatal suture expansion in rats. Their work consisted of 32 male Wistar rats and randomly divided into four groups. The Isotretinoin group was treated with 7.5 mg/kg of Isotretinoin, and the soybean group was treated with 2 mL/kg of soybean oil for 57 days. It was concluded that Isotretinoin had no negative effects on bone formation after maxillary suture expansion in rats.

Regarding the long-term effects on the stability of bone remodeling, with the use of retinoids, changes in bone biomechanical resistance may be included, making bones more susceptible to fractures. A study in senile rats that were fed high doses of retinoic acid for 12 weeks, using the mechanical flexion test, demonstrated that less Newton force (N) is required to cause fractures, indicating the negative effects of excess vitamin A intake [54].

Since this is a translational study, and due to the scarcity of research that analyzed the effect of Isotretinoin in conjunction with maxillary expansion, and that the adverse effects caused by the administration of Isotretinoin depend on the dosage, duration of treatment, or another animal model [15,26,50,51,53], it becomes evident that more research on the subject is needed. We can consider as one of the limitations of the study the absence of comparative groups with other dosages and periods of use of Isotretinoin. Therefore, the results of the current study should be interpreted considering this limitation. Furthermore, future studies should address this aspect to provide a more comprehensive understanding.

## 5. Conclusions

According to the data obtained in this study, it was concluded that the daily administration of Isotretinoin at the standard dose for the treatment of severe acne does not cause significant alterations in the bone repair process following the opening of the median palatine suture in rats. This is an important finding for orthodontists to know that, within the limitations of this preclinical study, Isotretinoin did not impair early bone healing during rapid maxillary expansion. However, due to the scarcity of research in this area, more studies are needed to fully evaluate its effects.

## Data Availability

Data presented in this study are available on request from the corresponding author.

## Supplementary Materials

The following supporting information can be downloaded at: https://www.mdpi.com/article/10.3390/dj13040142/s1, Table S1: Descriptive statistics and Shapiro–Wilk test of normality for the different measures.
