# Supplementary material for: Microtomographic, Histomorphological, and Histomorphometric Analysis of Bone Healing in the Midpalatal Suture After Treatment with Isotretinoin
Source: Dent J (Basel). 2025 Mar 25;13(4):142. doi: 10.3390/dj13040142 (PMC12025938; doi:10.3390/dj13040142)
Supplement: Supplementary file 1 [file dentistry-13-00142-s001.zip › dentistry-3494870-supplementary.pdf]

Table 1. Descriptive statistics and Shapiro-Wilk test of normality for the different measures

Parameter	Group	Period (days)	Descriptive Statistic				Confidence Intervals		Shapiro-Wilk	
			Min	Max	Mean	SD	Lower	Upper	W	P value
**Micro-CT**										
Intra-incisor distance (µm)	CG	0	1124	1539	1342	149.2	1186	1499	0.9735	0.9148
		7	1621	2034	1818	175.2	1634	2002	0.9087	0.4276
		14	1802	2637	2133	292.3	1827	2440	0.9414	0.6703
	IG	0	1133	1457	1294	117.7	1171	1418	0.9752	0.9252
		7	1382	2107	1784	238.2	1534	2034	0.9379	0.6421
		14	1662	2257	1895	254.9	1628	2163	0.8421	0.1358
Palatal suture width (µm)	CG	0	85.36	135.0	112.5	17.06	94.57	130.4	0.9739	0.9178
		7	175.4	338.1	254.0	55.21	196.1	312.0	0.9843	0.9708
		14	265.1	509.8	371.8	91.70	275.6	468.1	0.9484	0.7273
	IG	0	82.41	133.6	105.8	17.56	87.40	124.3	0.9848	0.9726
		7	129.0	321.2	222.6	71.35	147.7	297.4	0.9517	0.7542
		14	211.7	395.4	297.4	61.78	232.6	362.2	0.9822	0.9619
**Histology**										
Midpalatal suture a width (µm)	CG	0	80.35	130.0	106.2	17.46	87.89	124.5	0.9721	0.9064
		7	185.1	346.2	267.9	63.37	201.4	334.4	09497	0.7375
		14	392.0	609.4	487.0	76.01	407.3	566.8	0.9750	0.9239
	IG	0	84.26	133.5	109.3	18.06	90.32	128.2	0.9383	0.6454
		7	170.2	318.6	257.8	63.28	191.4	324.2	0.8437	0.1397
		14	327.7	515.3	408.3	70.60	334.2	482.4	0.9445	0.6955
Bone volume density in midpalatal suture (%)	CG	0	-	-	-	-	-	-		
		7	11.54	31.15	19.82	7.235	12.23	27.41	0.9521	0.8506
		14	38.94	57.31	51.29	6.605	44.36	58.22	0.7574	0.1591
	IG	0	-	-	-	-	-	-		
		7	12.85	31.74	22.99	6.819	15.83	30.14	0.9781	0.9418
		14	41.86	59.35	52.63	6.407	45.90	59.35	0.9328	0.6020
Volume density of soft tissue in midpalatal suture (%)	CG	0	-	-	-	-	-	-		
		7	68.85	88.46	80.18	7.2335	75.59	87.77	0.9521	0.7574
		14	42.69	61.06	48.71	6.605	41.78	55.64	0.8506	0.1591
	IG	0	-	-	-	-	-	-		
		7	68.26	87.15	77.02	6.819	69.86	84.17	0.9781	0.9418
		14	40.65	58.14	47.38	6.407	40.65	54.10	0.9328	0.6020
Volume density of vessels in the soft tissue (%)	CG	0	2.680	5.490	4.127	1.087	2.986	5.267	0.9515	0.7521
		7	2.850	9.200	6.503	2.641	3.732	9.275	0.9044	0.4008
		14	5.050	17.55	10.33	4.576	5.523	15.13	0.9539	0.7714
	IG	0	3.960	6.730	4.980	1.035	3.894	6.066	0.9162	0.4783
		7	3.660	6.120	5.193	0.9290	4.218	6.168	0.9136	0.4606
		14	4.120	13.88	9,167	3.328	5.071	12.06	0.9756	0.9275
**Picrosirus-red/polarization**										
Total Birefringence/ collagen fibers (x10^6 pixels^2)	CG	0	100.81	122.11	113.06	9.26	103.33	122.79	0.8540	0.1697
		7	29.40	54.97	40.604	8.47	31.70	49.50	0.9487	0.7295
		14	51.45	76.32	59.97	9.03	50.49	69.46	0.8797	0.2677
	IG	0	94.28	119.18	108.24	10.22	97.51	118.98	0.9128	0.4553
		7	31.88	67.00	42.86	12.49	29.75	55.98	0.7985	0.0570
		14	44.43	80.37	64.37	15.17	48.45	80.29	0.8882	0.3089
Green birefringence (x10^6 pixels^2)	CG	0	30.69	41.82	36.34	4.31	31.81	40.87	0.9411	0.6684
		7	18.48	30.24	23.68	4.62	18.82	28.53	0.9327	0.6010
		14	26.05	45.35	36.13	6.91	28.87	43.39	0.9929	0.9951
	IG	0	20.90	49.11	33.39	9.01	23.93	42.85	0.8356	0.1199

		7	18.46	30.92	22.67	4.41	18.04	27.31	0.8595	0.1874
		14	26.43	44.61	35.13	6.62	28.17	42.09	0.9795	0.9492
Yellow birefringence (x10^6 pixels^2)	CG	0	22.64	43.39	31.13	7.94	22.79	39.47	0.9254	0.5449
		7	2.74	5.26	3.92	0.93	2.94	4.91	0.9630	0.8423
		14	4.71	7.05	5.61	1.03	4.53	6.69	0.8293	0.1060
	IG	0	29.09	36.00	31.91	2.57	29.22	34.61	0.9443	0.6939
		7	2.69	6.48	4.10	1.31	2.73	5.48	0.8976	0.3602
		14	3.88	8.87	6.47	1.85	4.53	8.42	0.9806	0.9543
Red birefringence (pixels^2)	CG	0	41.92	51.17	45.58	3.92	41.46	49.69	0.8659	0.2105
		7	3.43	20.37	12.02	6.38	5.32	18.72	0.9654	0.8604
		14	11.73	28.28	20.24	5.92	14.02	26.45	0.9361	0.6282
	IG	0	33.64	51.01	42.93	7.06	35.52	50.34	0.9290	0.5726
		7	9.52	29.59	16.08	7.09	8.63	23.53	0.8267	0.1008
		14	14.11	34.94	22.75	8.31	14.03	31.48	0.9264	0.5525
